# The face of hypervirulent *Klebsiella pneumoniae* isolated from clinical samples of two Iranian teaching hospitals

**DOI:** 10.1186/s12941-021-00467-2

**Published:** 2021-08-31

**Authors:** Rahimeh Sanikhani, Mohammad Moeinirad, Hamid Solgi, Azar Hadadi, Fereshteh Shahcheraghi, Farzad Badmasti

**Affiliations:** 1 Department of Bacteriology, Pasteur Institute of Iran, Tehran, Iran; 2 Division of Bacteriology, Department of Pathobiology, School of Public Health, Tehran University of Medical Sciences, Tehran, Iran; 3 Division of Clinical Microbiology, Department of Laboratory Medicine, Amin Hospital, Isfahan University of Medical Sciences, Isfahan, Iran; 4 Sina Hospital, Internal Medicine Department, and Research Center for Clinical Virology, Tehran University of Medical Sciences, Tehran, Iran; 5 Microbiology Research Center (MRC), Pasteur Institute of Iran, Tehran, Iran

**Keywords:** Hypervirulent *Klebsiella pneumonia*, Identification test, Antimicrobial resistance, Clonal relatedness

## Abstract

Hypervirulent *Klebsiella pneumoniae* (hvKp) has emerged as a pathogen of global concern. In this study, both phenotypic and genotypic tests were used to detect hvKp. Antimicrobial resistance profiles and clonal relatedness of clinical isolates were also determined. We found that 34.2% (163/477) of the isolates were tellurite resistant, and among them 102 hvKp isolates detected with *iucA* or *iutA* or *peg-344* as molecular markers. The *bla*_SHV_ (80.4%), followed by *bla*_CTX-M-15_ (76.5%) and *bla*_TEM_ (67.6%), *bla*_OXA-48_ (53.9%), and *bla*_NDM-1_ (32.3%) were detected, while *bla*_KPC-1_ was not present in any hvKp isolates. It was found that the majority of hvKp isolates belonged to capsular serotype K20 and *ompK36* group C, which is related to clonal group (CG) 23 (e.g. ST23). A high percentage of multidrug-resistant hvKp (76.6%) and high resistance to imipenem (67%) indicated a serious problem that should be addressed in the clinical setting.

## Introduction

Hypervirulent *Klebsiella pneumoniae* (hvKp), an emerging pathotype of *K. pneumoniae* was first reported from Taiwan. It was identified as an important cause of pyogenic liver abscess [1, 2]. In hvKp isolates, pLVPK-like plasmids (Large Virulence Plasmid of *K. pneumoniae*) encoding virulence factor genes including capsular polysaccharide synthesis regulators (*rmpA* and *rmpA2*) and iron acquisition systems (*iucA*, *iutA*, and *iro* siderophore gene cluster), a metabolic transporter (*peg-344*) and also heavy metal resistance genes (copper, silver, lead, and tellurite), have been identified [3, 4]. Therefore, most hvKp isolates are able to reduce tellurite and form a black colony due to the presence of the major virulence plasmids containing a tellurite resistance gene [5]. The pLVPK-like plasmids may carry all virulence factor genes or have lost some of them [6, 7]. On the other hand, the acquisition of antibiotic resistance plasmids or insertion of resistant mobile genetic elements into the hvKp plasmid turns them into superbugs that can be termed hyper-resistant hvKp strains [8–10]. Some *K. pneumoniae* clones are characterized as high-risk clones that play an important role in the spread of antibiotic-resistant strains [11, 12]. The association of the porin *ompK36* with clonal relatedness of *K. pneumoniae* isolates has been described in several studies [13, 14]. This typing method can be considered as a rapid method for characterizing the clonal relatedness of *K. pneumoniae* isolates. Four different genotypes for *ompK36* porin (A to D) in *K. pneumoniae* were defined and the correlation of different variants of *ompK36* with specific sequence types (STs) was illustrated [13–15].

It is important to distinguish hvKps from classical *K. pneumoniae* (cKp) isolates. To date, several methods have been used to identify hvKp isolates. Detection of hypermucoviscous phenotype on agar (string test), use of *Galleria mellonella* infection model, serum killing assay and mouse infection models are some of the methods used for phenotypic identification of these strains [16, 17]. However, there is no consensus phenotypic test for the early diagnosis of hvKps, and it appears that more accurate phenotypic tests are needed to rapidly identify these pathotypes of *K. pneumoniae*. In addition to phenotypic methods, the presence of different virulence genes has been studied to increase the sensitivity and accuracy of hvKps identification. Among these, the genes of *iucA*, *iutA*, and *peg-344* have been introduced as the best genetic diagnostic markers with the highest accuracy [18].

Therefore, the aim of this study was to develop a rapid identification method for hypervirulent *K. pneumoniae*. We also investigated the genotypic characteristics, prevalence of virulence factors and antibiotic resistance of hvKps in clinical samples isolated from Iran.

## Materials and methods

### Bacterial isolation and identification

In this cross-sectional study, we collected a total of 477 non-repetitive *K. pneumoniae* as clinical isolates from two educational hospitals in Tehran over a period of time from June 2019 to December 2020. All bacterial isolates were identified using standard biochemical laboratory methods and then the isolates were stored in a freezer at −70 °C in nutrient broth containing 20% glycerol until further studies.

### HvKp phenotypic identification

#### Tellurite resistance

We used tellurite agar culture as a rapid screening test in this study. The isolates that formed black colonies on this tellurite-containing selective medium were considered as presumptive hypervirulent strains for further study. For this purpose, 0.1 g of potassium tellurite powder was first dissolved in 10 ml of sterile distilled water and filtered using membrane filters of pore size 0.45 µm. Then we added 300 μl of the potassium tellurite solution to 100 ml of Mueller–Hinton agar medium, which was autoclaved and cooled to 45–50 °C. Finally, we dispensed into sterile plates. Colonies were examined after overnight incubation at 37 °C.

#### String test

Hypermucoviscous phenotype of the hvKp isolates was examined by the string test, and the positive result was confirmed via the formation of a 5-mm viscous filament by stretching of bacterial colonies on a blood agar after 24 h of incubation at 37 °C [19].

### Molecular characteristics

#### DNA extraction and identification

Plasmid DNA extraction Mini Kit (FAVORGEN Biotech Corporation, Taiwan) has been used for the detection of genes carried on plasmids. In addition, the boiling method was used for isolation of genomic DNA [20]. All amplification reactions for PCR assays were prepared in a total volume of 25 μl. The list of primer sequences, PCR product sizes, and PCR conditions is shown in Table 1. Finally, all PCR amplification products were sequenced and then searched in the GenBank database using BLAST tool (http://www.ncbi.nlm.nih.gov/blast/).

**Table 1:** Primers, product sizes, and annealing temperatures used for this study

Primer name	Primer sequence (5´ → 3´)	Amplicon size (bp)	Annealing temperature (ºC)	Refs.
*iucA*	F: AATCAATGGCTATTCCCGCTG R: CGCTTCACTTCTTTCACTGACAGG	239	62	[18]
*peg-344*	F: GCGGGAAAGGACAGAAAGCCAGTG R: GAGGGAAGATGAGAAATACGAGC	332	56	This study
*iutA*	F: GCCGCTAGGTTGGTGATGT R: CTCTGGTCGTGCTGGTTGA	949	61	This study
*iroB*	F: GTGTTGGATTCCGCCAGTGA R: TTCCGCCGCTACCTCTTCA	366	61	This study
*magA*	F: GGTGCTCTTTACATCATTGC R: GCAATGGCCATTTGCGTTAG	1282	51	[47]
*rmpA*	F: GAGTATTGGTTGACAGCAGGAT R: AGCCGTGGATAATGGTTTACAA	250	53	This study
*kfu*	F: ATAGTAGGCGAGCACCGAGA R: AGAACCTTCCTCGCTGAACA	520	60	[48]
*allS*	F: CCGAAACATTACGCACCTTT R: ATCACGAAGAGCCAGGTCAC	508	60	[48]
*ybt*	F: GACGGAAACAGCACGGTAAA R: GAGCATAATAAGGCGAAAGA	242	60	[47]
*bla*_CTX-M-15_	F: CGCTTTGCGATGTGCAG R: ACCGCGATATCGTTGGT	590	53	This study
*bla*_TEM_	F: GAGTATTCAACATTTCCGTGTC R: TAATCAGTGAGGCACCTATCTC	800	54	[49]
*bla*_SHV_	F: AAGATCCACTATCGCCAGCAG R: ATTCAGTTCCGTTTCCCAGCGG	200	60	[49]
*bla*_OXA-48_	F-GCGTGGTTAAGGATGAACAC R-CATCAAGTTCAACCCAACCG	745	60	[49]
*bla*_KPC-1_	F: CGTCTAGTTCTGCTGTCTTG R:CTTGTCATCCTTGTTAGGCG	798	55	[50]
*bla*_NDM-1_	F: GGTTTGGCGATCTGGTTTTC R: CGGAATGGCTCATCACGATC	621	54	[50]
*ompk36* group A	F: GAAGGCGCTCTGTCTCCTA R: TGCCATCATAGATGTCATAGG	97	60	[13]
*ompk36* group B	F: CGGTCGTGGCGCGCAGAAA R: GGTTGTTCTGA TCGTCGGTA	125	64	[13]
*ompk36* group C	F: CAACAACGGTCGT GGTTGGA R: CCCAGTGCCGGAACACTATT	144	62	[13]
*ompk36* group D	F: GAAGGTACTTCTCCGACCAA R: AATCAGATTCTCCGTTGCCG	283	62	[13]

#### HvKp molecular identification

All tellurite-resistant *K. pneumoniae* were screened for the presence of the aerobactin (*iucA*), its receptor (*iutA*) genes and *peg-344*. The isolates containing the *iucA* or *iutA* or *peg-344* genes were considered as hvKps [18].

### Antimicrobial susceptibility testing for hvKp

Antimicrobial susceptibility testing was performed using the disc diffusion method according to the clinical and laboratory standards institute (CLSI) guidelines (CLSI 2018-M100-S28) by the following antibiotic discs including amikacin (AK), gentamicin (GN), cefotaxime (CTX), ceftazidime (CAZ), ceftriaxone (CRO), imipenem (IMI), meropenem (MRP), cefepime (FEP), ciprofloxacin (CIP), ampicillin (AMP) and aztreonam (AZM). Minimum inhibitory concentrations (MICs) of imipenem and ceftazidime were determined by broth dilution method. *Escherichia coli* ATCC 25922 was used as the quality control strain for antimicrobial susceptibility testing.

### Capsular genotyping and detection of virulence, and antimicrobial resistance genes

The hvKp capsular serotypes K1, K2, K5, K20, K54, and K57 were identified using PCR method [21]. The hvKp virulence genes including salmochelin siderophore (*iroB*), mucoviscosity-associated gene (*magA*), *Klebsiella* ferric uptake (*kfu*), yersiniabactin (*ybt*), allantoin metabolism gene (*allS*), and *rmpA* were detected by specific primers listed in Table 1. In addition, PCR assays were carried out for detection of *bla*_TEM_, *bla*_SHV_, and *bla*_CTX-M-15_, *bla*_KPC-1_, *bla*_NDM-1_, and *bla*_OXA-48_ genes in all hvKp isolates.

### Determination of clonal relatedness using *ompK36* typing

All hvKp isolates were subjected to *ompK36* typing by the PCR-based method described by Yan et al*.,* using four pairs of primers [13].

### Statistical analysis

The statistical analyses of data were performed using SPSS software, version 16.0 (IBM, Armonk, NY, USA) and Chi-square tests (2 × 2 contingency table) were used to compare the data associated with hvKp and cKp strains. Finally, the P values < 0.05 was considered statistically significant.

### Nucleotide accession numbers

The accession numbers of *bla*_OXA-48_, *bla*_NDM-1_, *iutA*, *iucA* and *peg-344* are MZ245618, MZ245619, MZ245620, MZ245621 and MZ245622, respectively in GenBank database.

## Results

### Phenotypic tests

In this study, 163 (34.2%) out of 477K*. pneumoniae* isolates were able to grow on tellurite-containing MH medium and were considered tellurite-resistant strains, so they were selected for the molecular identification test. In addition, 62 out of 477K*. pneumoniae* isolates (13%) were reported with positive string test and hypermucoviscous phenotype.

### Molecular identification of hvKp

Based on molecular identification, we found the *iucA* or *iutA* or *peg-344* as hvKp molecular markers in 21.4% (102/477) of total *K. pneumoniae* and 62.6% (102/163) of tellurite-resistant isolates. Therefor, 45 isolates had only the *iucA*, 6 isolates had only the *iutA*, and 48 strains had both the *iucA* and *iutA* genes, all three genes (*iucA*, *iutA* and *peg-344*) were detected simultaneously in only three hvKp isolates. Also, 48% (49/102) hvKp isolates were string positive. See Table 2.

**Table 2:** Demographic data of patients, phenotypic and molecular characteristics of the hvKp isolates included in this study

Hospital	Ward	Source	String test	Virulence factor genes	Capsule serotype	*ompK36* typing	Antibiotic resistance pattern	Antibiotic resistance genes	MIC (µg/ml) CAZ IMI
A	Internal	Tracheal	Neg	*iucA, iutA, ybt*	K20	C	CTX, GN, IMI, FEP, CRO, CAZ, CIP, MRP, AZM	*bla*_TEM_*, bla*_SHV_*, bla*_CTX-M-15_ and *bla*_OXA-48_	ND ND
A	ICU	Blood	Neg	*iucA, iutA, ybt*	K20	C	CTX, GN, IMI, FEP, CRO, CAZ, CIP, MRP, AZM	*bla*_TEM_*, bla*_SHV_*, bla*_CTX-M-15_ and *bla*_OXA-48_	ND ND
A	Emergency	Urine	Pos	*iucA, kfu, ybt*	ND	B	ND	*bla*_SHV_	ND ND
A	ICU	Tracheal	Neg	*iucA*	K20	A	CTX, GN, IMI, FEP, CRO, AK, CAZ, CIP, MRP, AZM	*bla*_TEM_*, bla*_SHV_*, bla*_OXA-48_ and *bla*_NDM-1_	ND ND
A	Surgery	Abscess	Pos	*iucA, ybt*	ND	A	CTX, GN, IMI, FEP, CRO, AK, CAZ, CIP, MRP, AZM	*bla*_TEM_*, bla*_SHV_*, bla*_CTX-M-15_*, bla*_OXA-48_ and *bla*_NDM-*1*_	ND ND
A	Surgery	Abscess	Pos	*iucA*	ND	C	ND	*bla*_SHV_	ND ND
A	Surgery	Abdominal Secretions	Pos	*peg-344, iucA, iutA, iro, ybt*	K20	C	CTX, GN, IMI, FEP, CRO, CAZ, CIP, MRP	*bla*_SHV_ and *bla*_CTX-M-15_	16 < 2
A	ICU	Tracheal	Neg	*iucA*	ND	A	CTX, GN, IMI, FEP, CRO, AK, CAZ, CIP, MRP, AZM	*bla*_TEM_*, bla*_SHV_*, bla*_OXA-48_ and *bla*_NDM-*1*_	ND ND
A	ICU	Urine	Pos	*iucA, kfu*	ND	B	Susceptible	ND	ND ND
A	ICU	Blood	Neg	*iucA, ybt*	ND	A	CTX	*bla*_SHV_	ND ND
A	ICU	Synovial fluid	Neg	*iucA, kfu*	ND	B	Susceptible	ND	ND ND
A	Out-patient	Urine	Pos	*iucA, ybt*	ND	C	GN, CTX, CAZ	*bla*_TEM_ and *bla*_SHV_	ND ND
A	ICU	Abscess	Neg	*iucA, iutA*	K20	C	CTX, GN, IMI, CRO, CAZ	*bla*_TEM_	ND ND
A	Internal	Urine	Neg	*iucA*	ND	A	ND	*bla*_TEM_ and *bla*_SHV_	ND ND
A	ICU	Urine	Pos	*iucA, kfu, ybt*	K2	C	CTX, GN, IMI, FEP, CRO, AK, CAZ, CIP, MRP, AZM	*bla*_TEM_*, bla*_SHV_*, bla*_CTX-M-15_ and *bla*_OXA-48_	ND ND
A	Internal	Urine	Neg	*iucA, ybt*	ND	D	ND	*bla*_SHV_*, bla*_CTX-M-15_ and *bla*_OXA-48_	ND ND
A	Out-patient	Urine	Pos	*iucA, ybt*	ND	C	Susceptible	ND	ND ND
A	Internal	Urine	Pos	*iucA, kfu, ybt*	ND	C	ND	*bla*_SHV_ and *bla*_CTX-M-15_	ND ND
A	Out-patient	Urine	Neg	*iucA, iutA*, *ybt, rmpA*	ND	C	CTX, GN, IMI, FEP, CRO, AK, CAZ, CIP, MRP, AZM	*bla*_TEM_*, bla*_CTX-M-15_ and *bla*_NDM-1_	ND ND
A	Out-patient	Urine	Pos	*iucA, kfu*	ND	A	ND	*bla*_TEM_*, bla*_SHV_ and *bla*_CTX-M-15_	ND ND
A	Out-patient	Urine	Pos	*iucA, ybt*	K20	B	ND	*bla*_SHV_ and *bla*_CTX-M15_	ND ND
A	Internal	Urine	Neg	*iucA, kfu*	ND	C	ND	*bla*_SHV_	ND ND
A	Internal	Sputum	Pos	*peg-344, iucA, iutA, ybt, rmpA*	K20	C	CTX, CAZ	ND	ND ND
A	Surgery	Abdominal Secretions	Pos	*iucA*	ND	A	ND	*bla*_SHV_, *bla*_OXA*-48*_ and *bla*_NDM-1_	ND ND
A	Emergency	Urine	Pos	*iucA, kfu, ybt*	K2	C	CTX, GN, IMI, FEP, CRO, CAZ, CIP, MRP, AZM	*bla*_TEM_*, bla*_SHV_*, bla*_CTX-M-15_ and *bla*_OXA-48_	ND ND
A	Out-patient	Urine	Pos	*iucA, kfu, ybt*	K2	C	CTX, GN, IMI, FEP, CRO, CAZ, CIP, MRP, AZM	*bla*_TEM_*, bla*_SHV_*, bla*_CTX-M-15_ and *bla*_OXA-48_	ND ND
A	Surgery	Urine	Pos	*iucA, kfu, ybt*	ND	A	CTX, GN, IMI, FEP, CRO, AK, CAZ, CIP, MRP, AZM	*bla*_TEM_*, bla*_SHV_*, bla*_CTX-M-15_*, bla*_OXA-48_ and *bla*_NDM-*1*_	ND ND
A	Emergency	Urine	Pos	*iucA, kfu*	ND	A	ND	*bla*_SHV_	ND ND
A	ICU	Blood	Pos	*iucA, kfu*	ND	D	CTX, GN, IMI, FEP, CRO, AK, CAZ, CIP, MRP, AZM	*bla*_SHV_*, bla*_OXA-48_ and *bla*_NDM-1_	ND ND
A	ICU	Wound	Neg	*iucA, ybt*	ND	D	Susceptible	ND	ND ND
A	Graft	Urine	Neg	*iucA, kfu*, *rmpA*	K20	C	CTX, GN, IMI, FEP, CRO, CAZ, CIP, MRP	*bla*_TEM_*, bla*_CTX-M-15_ and *bla*_OXA-48_	ND ND
A	Out-patient	Urine	Pos	*iucA, kfu*	ND	C	CTX, GN, FEP, CRO, CAZ, CIP	*bla*_TEM_*, bla*_SHV_ and *bla*_CTX-M15_	ND ND
A	Graft	Urine	Neg	*iucA, ybt, allS, rmpA*	K20	C	CTX, GN, IMI, FEP, CRO, CAZ, CIP, MRP, AZM	*bla*_TEM_*, bla*_SHV_*, bla*_CTX-M-15_*, bla*_OXA-48_*, bla*_NDM-*1*_	> 16 4
A	ICU	Abscess	Neg	*iucA*	ND	C	Susceptible	ND	ND ND
A	ICU	Urine	Neg	*iucA*	ND	C	CTX, FEP, CAZ	*bla*_SHV_	ND ND
A	ICU	Blood	Neg	*iucA*	ND	D	ND	*bla*_CTX-M-15_ and *bla*_OXA-48_	ND ND
A	ICU	Abscess	Pos	*iucA, iutA, ybt, rmpA*	K20	C	ND	*bla*_TEM_*, bla*_CTX-M15_ and *bla*_NDM-1_	64 > 4
A	Internal	Tracheal	Neg	*iucA, ybt*	ND	A	CTX, GN, IMI, FEP, CRO, AK, CAZ, CIP, MRP, AZM	*bla*_SHV_*, bla*_CTX-M-15_ and *bla*_OXA-48_	ND ND
A	ICU	Tracheal	Neg	*iucA, iutA*	ND	C	CTX, GN, FEP, CRO, AK, CAZ	*bla*_SHV_	ND ND
A	Surgery	Wound	Neg	*iucA, kfu, ybt*	ND	C	CTX, CRO, CIP	*bla*_SHV_	ND ND
A	Internal	Abscess	Neg	*iucA, ybt*	ND	D	CTX, GN, IMI, CRO, CAZ, CIP, MRP, AZM	*bla*_TEM_*, bla*_SHV_*, bla*_CTX-M-15_ and *bla*_OXA-48_	ND ND
A	Surgery	Abscess	Neg	*iucA, ybt*	ND	C	CTX, GN, IMI, FEP, CRO, AK, CAZ, CIP, MRP, AZM	*bla*_TEM_*, bla*_CTX-M-15_ and *bla*_OXA-48_	ND ND
A	Emergency	Urine	Neg	*iucA, kfu, ybt*	ND	C	Susceptible	ND	ND ND
A	Internal	Sputum	Pos	*iucA, iutA, ybt*	K20	C	CTX, GN, FEP, CRO, CAZ	*bla*_TEM_*, bla*_SHV_ and *bla*_CTX-M-15_	ND ND
A	Out-patient	Urine	Neg	*iucA, kfu, ybt*	ND	A	CTX, GN, IMI, FEP, CRO, AK, CAZ, CIP, MRP	*bla*_CTX-M-15_ and *bla*_OXA-48_	ND ND
A	ICU	Abscess	Pos	*iucA, ybt*	ND	D	CTX, GN, IMI, FEP, CRO, AK, CAZ, CIP, MRP, AZM	*bla*_SHV_*, bla*_CTX-M-15_*, bla*_OXA-48_ and *bla*_NDM-1_	> 16 8
A	Emergency	Blood	Neg	*iucA, kfu*	ND	B	AK	ND	ND ND
A	ICU	Tracheal	Neg	*iucA, ybt*	ND	A	CTX, GN, IMI, FEP, CRO, AK, CAZ, CIP, MRP, AZM	*bla*_CTX-M-15_ and *bla*_OXA-48_	ND ND
A	ICU	Blood	Pos	*iucA, ybt*	ND	D	CTX, GN, IMI, FEP, CRO, AK, CAZ, CIP, MRP, AZM	*bla*_SHV_*, bla*_CTX-M-15_ and *bla*_OXA-48_	ND ND
A	Neurology	Urine	Pos	*iucA, iutA, ybt, rmpA*	K20	C	CTX, GN, IMI, FEP, CRO, CAZ, CIP, MRP, AZM	*bla*_TEM_*, bla*_SHV_*, bla*_CTX-M-15_ and *bla*_OXA-48_	ND ND
A	Emergency	Urine	Pos	*iucA, iutA, ybt, rmpA*	K20	C	CTX, GN, IMI, FEP, CRO, CAZ, CIP, MRP, AZM	*bla*_TEM_*, bla*_SHV_*, bla*_CTX-M-15_ and *bla*_OXA-48_	ND ND
A	Internal	Wound	Pos	*iucA, iutA, ybt, rmpA*	K20	C	CTX, GN, IMI, FEP, CRO, AK, CAZ, CIP, MRP, AZM	*bla*_TEM_*, bla*_SHV_*, bla*_CTX-M-15_ and *bla*_OXA-48_	16 2
A	Emergency	Urine	Pos	*iucA, iutA, ybt, rmpA*	ND	C	CTX, GN, IMI, FEP, CRO, CAZ, CIP, MRP, AZM	*bla*_TEM_*, bla*_SHV_*, bla*_CTX-M-15_ and *bla*_OXA-48_	32 > 4
A	ICU	BAL	Pos	*iucA, iutA, ybt, rmpA*	K20	C	CTX, GN, IMI, FEP, CRO, CAZ, CIP, MRP, AZM	*bla*_TEM_*, bla*_SHV_*, bla*_CTX-M-15_ and *bla*_OXA-48_	ND ND
A	Surgery	Urine	Pos	*iucA, iutA, ybt, rmpA*	K20	C	CTX, GN, FEP, CRO, CAZ, CIP, MRP	*bla*_TEM_*, bla*_SHV_ and *bla*_CTX-M15_	ND ND
A	ICU	Blood	Pos	*iucA, iutA, ybt, rmpA*	K20	C	CTX, GN, IMI, FEP, CRO, AK, CAZ, CIP, MRP, AZM	*bla*_TEM_*, bla*_SHV_ and *bla*_CTX-M15_	> 16 > 4
A	Internal	Urine	Pos	*iucA, iutA, ybt, rmpA*	K20	C	CTX, GN, FEP, CRO, CAZ, CIP	*bla*_TEM_*, bla*_SHV_ and *bla*_CTX-M-15_	ND ND
A	ICU	Tracheal	Pos	*iucA, iutA, rmpA*	ND	C	CTX, GN, IMI, FEP, CRO, CAZ, CIP, MRP, AZM	*bla*_TEM_*, bla*_SHV_*, bla*_CTX-M-15_ and *bla*_OXA-48_	ND ND
A	ICU	BAL	Pos	*iucA, iutA, ybt, rmpA*	K20	D	CTX, GN, IMI, FEP, CRO, CAZ, CIP, MRP, AZM	*bla*_TEM_*, bla*_CTX-M-15_ and *bla*_OXA-48_	64 > 4
A	Emergency	Urine	Pos	*iucA, iutA, ybt*	K20	C	CTX, GN, IMI, FEP, CRO, CAZ, CIP, MRP	*bla*_TEM_ and *bla*_CTX-M15_	ND ND
A	ICU	CSF	Pos	*iucA, iutA, ybt, rmpA*	K20	C	CTX, GN, FEP, CRO, CAZ, CIP	*bla*_TEM_*, bla*_SHV_ and *bla*_CTX-M-15_	ND ND
A	ICU	Tracheal	Pos	*iucA, ybt*	K20	D	CTX, GN, IMI, FEP, CRO, CAZ, CIP, MRP, AZM	*bla*_TEM_*, bla*_SHV_*, bla*_CTX-M-15_ and *bla*_OXA-48_	ND ND
A	ICU	Blood	Pos	*iucA, iutA, ybt, rmpA*	K20	C	CTX, FEP, CRO, CAZ, CIP	*bla*_TEM_*, bla*_SHV_ and *bla*_CTX-M-15_	ND ND
B	Infectious	Sputum	Neg	*IutA, ybt*	ND	C	CTX, GN, FEP, CRO, CAZ, CIP	*bla*_CTX-M-15_	ND ND
B	Infectious	Sputum	Pos	*iucA, ybt*	ND	D	CTX, GN, IMI, FEP, CRO, CAZ, CIP, MRP, AZM	*bla*_TEM_*, bla*_SHV_*, bla*_CTX-M-15_ and *bla*_OXA-48_	64 8
B	ICU	Urine	Neg	*iucA, iutA, ybt, rmpA*	K20	C	CTX, GN, IMI, FEP, CRO, CAZ, CIP, MRP, AZM	*bla*_TEM_*, bla*_SHV_*, bla*_CTX-M-15_*, bla*_OXA-48_ and *bla*_NDM-*1*_	64 16
B	Infectious	Urine	Neg	*iucA, iutA, ybt, rmpA*	K20	C	CTX, GN, IMI, FEP, CRO, CAZ, CIP, MRP, AZM	*bla*_TEM_*, bla*_SHV_*, bla*_CTX-M-15_*, bla*_OXA-48_ and *bla*_NDM-*1*_	> 16 > 4
B	ICU	Tracheal	Neg	*iucA, iutA, ybt, rmpA*	K20	C	CTX, GN, IMI, FEP, CRO, CAZ, CIP, MRP, AZM	*bla*_TEM_*, bla*_SHV_*, bla*_CTX-M-15_*, bla*_OXA-48_ and *bla*_NDM-*1*_	64 4
B	ICU	Tracheal	Pos	*iucA, iutA, ybt, rmpA*	K20	C	CTX, GN, IMI, FEP, CRO, AK, CAZ, CIP, MRP, AZM	*bla*_TEM_*, bla*_SHV_*, bla*_CTX-M-15_ and *bla*_OXA-48_	64 > 4
B	ICU	Urine	Neg	*iucA, iutA, ybt, rmpA*	K20	C	CTX, GN, FEP, CRO, CAZ, CIP, AZM	*bla*_TEM_*, bla*_SHV_ and *bla*_CTX-M-15_	ND < 2
B	ICU	Tracheal	Neg	*iucA, kfu, ybt*	K20	C	CTX, GN, IMI, FEP, CRO, AK, CAZ, CIP, MRP, AZM	*bla*_TEM_*, bla*_SHV_*, bla*_CTX-M-15_ and *bla*_NDM-1_	> 16 > 4
B	ICU	Tracheal	Neg	*iucA, iutA, ybt, rmpA*	K20	C	CTX, GN, IMI, FEP, CRO, AK, CAZ, CIP, MRP, AZM	*bla*_TEM_*, bla*_SHV_*, bla*_CTX-M-15_*, bla*_OXA-48_ and *bla*_NDM-*1*_	64 4
B	Surgery	Wound	Neg	*iucA, iutA, ybt, rmpA*	K20	C	CTX, FEP, CRO, AK, CAZ, CIP	*bla*_TEM_*, bla*_SHV_ and *bla*_CTX-M-15_	ND ND
B	ICU	Blood	Pos	*peg-344, IucA, iutA, iro, kfu, ybt, magA, allS*	K1	C	CTX, FEP, CRO, CAZ, CIP	*bla*_TEM_*, bla*_SHV_ and *bla*_CTX-M-15_	> 16 < 2
B	ICU	Tracheal	Neg	*iucA, ybt, rmpA*	K20	C	CTX, FEP, CRO, CAZ, CIP	*bla*_TEM_*, bla*_SHV_*, bla*_CTX-M-15_	ND 2
B	ICU	Tracheal	Neg	*iucA, iutA, ybt, rmpA*	K20	C	CTX, FEP, CRO, CAZ, CIP	*bla*_TEM_*, bla*_SHV_ and *bla*_CTX-M-15_	ND 2
B	ICU	Urine	Neg	*iucA, iutA, ybt, rmpA*	K20	C	CTX, FEP, CRO, CAZ, CIP	*bla*_TEM_*, bla*_SHV_*, bla*_CTX-M-15_	ND 2
B	Surgery	Urine	Pos	*iucA, iutA, ybt, rmpA*	K20	C	CTX, FEP, CRO, CAZ, CIP	*bla*_TEM_*, bla*_SHV_ and *bla*_CTX-M-15_	ND ND
B	ICU	Tracheal	Pos	*iutA, iro, rmpA*	ND	C	CTX, GN, FEP, CRO, AK, CAZ, CIP	*bla*_CTX-M-15_	ND ND
B	ICU	Tracheal	Neg	*iutA*	ND	C	CTX, GN, IMI, FEP, CRO, CAZ, CIP, MRP	*bla*_TEM_ and *bla*_SHV_	ND ND
B	Internal	Urine	Pos	*iucA, iutA, ybt, rmpA*	K20	C	CTX, GN, IMI, FEP, CRO, CAZ, CIP, MRP	*bla*_TEM_*, bla*_SHV_*, bla*_CTX-M-15_ and *bla*_OXA-48_	ND 4
B	Internal	Tracheal	Pos	*iucA, iutA, kfu, ybt, rmpA*	K20	D	CTX, GN, IMI, FEP, CRO, CAZ, CIP, MRP	*bla*_TEM_*, bla*_SHV_*, bla*_CTX-M-15_ and *bla*_OXA-48_	ND > 4
B	Emergency	Urine	Pos	*iucA, iutA, kfu, ybt, rmpA*	K20	C	CTX, FEP, CRO, AK, CAZ, CIP	*bla*_SHV_ and *bla*_CTX-M-15_	ND ND
B	Surgery	Tracheal	Neg	*iucA, iutA, ybt, rmpA*	K20	C	CTX, GN, FEP, CRO, CAZ, CIP	*bla*_SHV_ and *bla*_CTX-M-15_	ND ND
B	ICU	Tracheal	Pos	*iucA, iutA, ybt, rmpA*	K20	C	CTX, GN, IMI, FEP, CRO, CAZ, CIP, MRP, AZM	*bla*_TEM_*, bla*_SHV_*, bla*_CTX-M15_*, bla*_OXA-48_ and *bla*_NDM-*1*_	32 > 4
B	Infectious	Tracheal	Neg	*iutA, ybt*	ND	A	CTX, GN, IMI, FEP, CRO, AK, CAZ, CIP, MRP, AZM	*bla*_TEM_*, bla*_SHV_*, bla*_CTX-M-15_*, bla*_OXA-48_ and *bla*_NDM-*1*_	64 > 4
B	ICU	Tracheal	Neg	*iucA, ybt, rmpA*	K20	C	CTX, GN, IMI, FEP, CRO, AK, CAZ, CIP, MRP, AZM	*bla*_TEM_*, bla*_SHV_*, bla*_CTX-M-15_*, bla*_OXA-48_ and *bla*_NDM-*1*_	32 4
B	ICU	Tracheal	Neg	*iutA*	K20	A	CTX, GN, IMI, FEP, CRO, AK, CAZ, CIP, MRP, AZM	*bla*_TEM_*, bla*_SHV_*, bla*_CTX-M-15_ and *bla*_NDM-*1*_	ND > 4
B	ICU	Tracheal	Neg	*iucA, ybt, rmpA*	K20	C	CTX, GN, IMI, FEP, CRO, AK, CAZ, CIP, MRP, AZM	*bla*_TEM_*, bla*_SHV_*, bla*_CTX-M-15_*, bla*_OXA-48_ and *bla*_NDM-*1*_	64 > 4
B	ICU	Tracheal	Neg	*iucA, iutA, ybt, rmpA*	K20	C	CTX, GN, IMI, FEP, CRO, AK, CAZ, CIP, MRP, AZM	*bla*_TEM_*, bla*_SHV_*, bla*_CTX-M-15_*, bla*_OXA-48_ and *bla*_NDM-*1*_	ND < 1
B	ICU	Tracheal	Neg	*iucA, iutA, ybt, rmpA*	K20	C	CTX, GN, IMI, FEP, CRO, CAZ, CIP, MRP, AZM	*bla*_TEM_*, bla*_SHV_*, bla*_CTX-M-15_*, bla*_OXA-48_ and *bla*_NDM-*1*_	ND 16
B	ICU	CSF	Neg	*iucA, iutA, ybt, rmpA*	K20	C	CTX, GN, IMI, FEP, CRO, CAZ, CIP, MRP, AZM	*bla*_TEM_*, bla*_SHV_*, bla*_CTX-M-15_*, bla*_OXA-48_ and *bla*_NDM-*1*_	128 > 4
B	ICU	Urine	Neg	*iucA, iutA, ybt, rmpA*	K20	C	CTX, GN, IMI, FEP, CRO, CAZ, CIP, MRP, AZM	*bla*_TEM_*, bla*_SHV_*, bla*_CTX-M-15_*, bla*_OXA-48_ and *bla*_NDM-*1*_	64 < 4
B	ICU	BAL	Pos	*iucA, iutA, ybt, rmpA*	K20	C	CTX, GN, IMI, FEP, CRO, AK, CAZ, CIP, MRP, AZM	*bla*_TEM_*, bla*_SHV_*, bla*_CTX-M-15_*, bla*_OXA-48_ and *bla*_NDM-*1*_	ND 4
B	ICU	Tracheal	Neg	*iucA, iutA, ybt, rmpA*	K20	C	CTX, GN, IMI, FEP, CRO, CAZ, CIP, MRP, AZM	*bla*_TEM_*, bla*_SHV_*, bla*_CTX-M-15_*, bla*_OXA-48_ and *bla*_NDM-*1*_	64 > 4
B	ICU	Tracheal	Pos	*iucA, iutA, ybt, rmpA*	K20	C	CTX, GN, IMI, FEP, CRO, CAZ, CIP, MRP, AZM	*bla*_TEM_*, bla*_SHV_*, bla*_CTX-M-15_*, bla*_OXA-48_ and *bla*_NDM-*1*_	ND > 4
B	Surgery	Blood	Neg	*iucA, iutA, ybt, rmpA*	K20	C	CTX, GN, IMI, FEP, CRO, AK, CAZ, CIP, MRP, AZM	*bla*_TEM_*, bla*_SHV_*, bla*_CTX-M-15_*, bla*_OXA-48_ and *bla*_NDM-*1*_	ND 256
B	ICU	Tracheal	Neg	*iucA, iutA, ybt, rmpA*	K20	C	CTX, GN, IMI, FEP, CRO, CAZ, CIP, MRP, AZM	*bla*_TEM_*, bla*_SHV_*, bla*_CTX-M-15_*, bla*_OXA-48_ and *bla*_NDM-*1*_	ND 16
B	Surgery	Wound	Neg	*iucA, iutA, ybt, rmpA*	K20	C	CTX, GN, IMI, FEP, CRO, CAZ, CIP, MRP, AZM	*bla*_TEM_*, bla*_SHV_*, bla*_CTX-M-15_*, bla*_OXA-48_ and *bla*_NDM-*1*_	ND ND
B	ICU	Tracheal	Pos	*iucA, iutA, iro, ybt, rmpA*	K20	C	CTX, GN, IMI, FEP, CRO, AK, CAZ, CIP, MRP, AZM	*bla*_TEM_*, bla*_SHV_*, bla*_CTX-M-15_*, bla*_OXA-48_ and *bla*_NDM-*1*_	ND 8
B	ICU	Tracheal	Neg	*iucA, iutA, ybt, rmpA*	K20	C	CTX, GN, IMI, FEP, CRO, CAZ, CIP, MRP, AZM	*bla*_TEM_*, bla*_SHV_*, bla*_CTX-M-15_*, bla*_OXA-48_ and *bla*_NDM-*1*_	ND 8
B	ICU	Tracheal	Neg	*iucA, iutA, ybt, rmpA*	K20	C	CTX, GN, IMI, FEP, CRO, CAZ, CIP, MRP, AZM	*bla*_SHV_*, bla*_CTX-M-15_*, bla*_OXA-48_ and *bla*_NDM-*1*_	ND 8

^*^M, male; F, female; ICU, Intensive Care Unit; BAL, bronchoalveolar lavage; CSF, cerebrospinal fluid; Pos, positive; Neg, negative; *iucA* and *iutA*, aerobactin genes; *ybt*, yersiniabactin; *rmpA*, regulator of mucoid phenotype; *Kfu*, Klebsiella iron uptake; *magA*, mucoviscosity-associated gene; *iroB*, salmochelin iron uptake systems; *allS*, allantoin metabolism gene; *peg-344*, metabolic transporter; AK, amikacin; GN, gentamicin; CTX, cefotaxime; CAZ, ceftazidime; CRO, ceftriaxone; IMI, imipenem; MRP, meropenem; FEP, cefepime; CIP, ciprofloxacin;; AZM, aztreonam; MIC, minimum inhibitory concentration; ND, not determined

### Demographic data of hvKp isolates

In this collection, 61.7% (63/102) and 38.2% (39/102) of hvKp strains were isolated from hospital A and B, respectively. Most of hvKp isolates were obtained from urine (33.3%, 34/102), followed by tracheal aspiration (27.5%, 28/102), blood (9.8%, 10/102) and abscess (7.8%, 8/102) specimens. Also among hospital wards, most hvKps were obtained from patients admitted to intensive care unit (ICU) (51%), internal (12.7%), surgical (10.8%) and emergency (8.8%) wards. Sixty-two patients (60.8%) were male. Most of patients were over 60 years of age (45.1%). See Table 2.

### Antimicrobial susceptibility testing

In this study, we investigated the antimicrobial susceptibility profile in 90 hvKp isolates. Susceptibility profiles against antimicrobials agents are shown in Table 2. The highest rate of antibiotic resistance was related to ampicillin (100%), followed by cefotaxime and ceftazidime (91%). The lowest rate of resistance was found in amikacin (27.8%). The resistance rates to other antibiotics including ceftriaxone, cefepime, ciprofloxacin, gentamicin, meropenem, imipenem and aztreonam were 87.7%, 86.6%, 84.4%, 78.8%, 67.7%, 67.7% and 58.8%, respectively. In addition, 76.6% of the isolates (69/90) were resistant to at least three classes of antibiotics and were defined as multidrug resistant (MDR) [21]. Finally, the MIC of some resistant hvKp (resistant in disk diffusion) to the antibiotics ceftazidime and imipenem was determined by broth dilution method. In accordance with the facilities and funding, 23 isolates resistant to imipenem and ceftazidime were selected for the MIC test. All 23 isolates selected for ceftazidime had MIC ≥ 16: for 6 isolates MIC ˃ 16, 2 isolates MIC = 16, 3 isolates MIC = 32, 11 isolates MIC = 64, one isolate MIC = 128. Of the 39 resistant hvKp isolates, 79.5% had a MIC above the CLSI resistance criteria for imipenem (MIC ≥ 4): for 16 isolates MIC ˃ 4, 6 isolates MIC = 4, 5 isolates MIC = 8, 3 isolates MIC = 16, one isolate MIC = 256. 4 isolates (10.2%) were classified as intermediate resistant (MIC = 2) and 4 isolates (7.8%) were classified as susceptible (MIC < 2).

### Capsular genotyping and detection of virulence genes, and antimicrobial resistance genes

Capsular genotyping (K genotyping) of hvKp isolates showed that capsular serotype K20 was detected in more than half of the hvKp strains (54.9%). K2 and K1 were identified in only 3 (2.9%) and one isolate (1%), respectively, while K5, K54 and K57 were not detected in any of the hvKp isolates. In addition, 42 isolates (41.2%) did not belong to serotypes K1, K2, K5, K20, K54 and K57 as shown in Table 2.

PCR for virulence-associated genes revealed that *ybt* (77.5%) was the most common virulence factor gene after *iucA*. The other virulence factor genes including *rmpA*, *iroB*, *magA*, *kfu* and *allS* were detected in 48%, 3.9%, 0.98, 21.6% and 1.96% hvKp isolates, respectively. See Table 2.

The distribution of ESBLs and carbapenemase genes among hvKp isolates are shown in Table 2. The results showed that 92.2% (94/102) of hvKp isolates carried at least one antibiotic resistance gene and only 7.8% (8/102) had no resistance gene. The *bla*_SHV_ was the most common Beta-lactamase gene (80.4%), followed by *bla*_CTX-M-15_ (76.5%) and *bla*_TEM_ (67.6%). Also, PCR amplification of carbapenemase genes showed that *bla*_OXA-48_ (53.9%) was the dominant genotype of carbapenem-resistant strains, followed by *bla*_NDM-1_ (32.3%), while *bla*_KPC-1_ was not detected in any hvKp isolate. Thus, 56.8% (58/102) of the hvKp isolates that co-carried *bla*_TEM_, *bla*_SHV_, *bla*_CTX-M-15_, *bla*_OXA-48_ and *bla*_NDM-1_ genes was the predominant MDR-hvKp genotype. On the other hand, as shown in Table 2, more than 2 virulence factor genes were detected simultaneously in the majority of resistant hvKp strains and virulence profiles including: *iucA, iutA, ybt* and *rmpA* genes have been reported in 40.4% (38/94) resistant hvKp isolates.

### ompK36 typing

PCR-based *ompK36* typing revealed that *ompK36* group C was the most common type with 70.6% (72/102) frequency. The prevalence of the other types, including *ompK36* groups A, B, and C, was (14/102) 13.7%, (5/102) 4.9%, and 10.8% (11/102), respectively. See Table 2.

### Comparison between cKps and hvKps

In this study, 78 cKps and 102 hvKps isolates were examined. Demographic data and antimicrobial resistance profile were compared using chi-square tests. The data showed that there were no significant differences in demographic data between two groups. However, significant differences were found in antimicrobial resistance (e.g. amikacin, cefotaxime and gentamicin) and the presence of carbapenemases (*bla*_OXA-48_, *bla*_NDM-1_). See Table 3.

**Table 3:** Comparison between classical *K. pneumoniae* (cKp) and hypervirulent *K. pneumoniae* (hvKp) in demographic data and antimicrobial resistance profile based on chi-square test

Variable^a^	Classic *K. pneumoniae* (n = 78)	Hypervirulent *K. pneumoniae* (n = 102)	P value^b^
Gender	45 (M) 33 (F)	62 (M) 40 (F)	0.67
Age ≥ 50	49	63	0.59
ICU	38	52	0.76
Trachea	20	28	0.49
Urine	22	36	0.31
Blood	7	10	0.85
Wound	4	5	0.94
CSF	1	2	0.72
Sputum	2	4	0.61
Amikacin resistance	36	25	**0.013**^**b**^
Cefotaxime resistance	78	82	**0.028**^**b**^
Gentamicin resistance	45	71	**0.003**^**b**^
Ceftazidime resistance	75	82	0.18
Cefepime resistance	73	78	0.13
Ciprofloxacin resistance	67	76	0.79
Imipenem resistance	64	61	0.052
*bla*_SHV_	63	83	0.91
*bla*_TEM_	60	69	0.17
*bla*_CTX-M-15_	66	77	0.13
*bla*_OXA-48_	24	55	**0.0019**^**b**^
*bla*_NDM-1_	50	33	**0.00002**^**b**^

^a^M, male; F, female; ICU, Intensive Care Unit; CSF, cerebrospinal fluid

^b^The bolded variable showed a significant difference

## Discussion

In this study, to identify these *K. pneumoniae* superbugs, we used a combination of phenotypic and genotypic methods including tellurite resistance, and preferential gene markers. Previously, only the string test was used as a phenotypic method to identify hvKp isolates, but the string test is not a reliable rapid test for hvKp detection [1, 22, 23]. MacConkey inositol potassium tellurite agar (MCIK) has been used as a selective medium for the detection of *K. pneumoniae* from environmental sources or animal and human fecal samples [24]. This study showed that the trait of tellurite resistance is strongly associated with CG23, CG65 and CG86, which are mostly invasive community-acquired strains of *K. pneumoniae*. It appears that the large virulence plasmids of hvKp harbor tellurium resistance genes [24]. In silico analysis revealed that the tellurium gene cluster is highly prevalent among hypervirulent plasmids which is present in different sequence types (data not yet published). This prompted us to use Mueller–Hinton agar containing potassium tellurite as a selective medium for the rapid detection of hypervirulent *K. pneumoniae* strains. In this study, out of 163 tellurite-resistant isolates, 102 strains were genetically confirmed as hypervirulent *K. pneumoniae*, so making this method superior than string test for rapid phenotypic hvKp identification.

We also used three key virulence genes as molecular biomarkers previously introduced by Russo et al. to increase the accuracy and sensitivity of hvKp detection [18]. In addition, all hvKp isolates were examined for the presence of other virulence factor genes. In general, the frequencies of virulence factor genes, from highest to lowest, *iucA*, *ybt*, *iutA*, *rmpA*, *kfu*, *iroB*, *peg-344*, *allS*, and *magA*, respectively, were reported. Other studies have also shown that the aerobactin is produced by more than 90% of hvKp, whereas only 6% of cKp strains can express it [18, 25]. In a study by XU et al. the prevalence of *iucA*, *iutA*, *rmpA* and *iro* was reported to be 56.8%, 56.8%, 43.2% and 40.9%, respectively. The prevalence of *iutA* and *rmpA* was similar to our study, but in the present study, the prevalence of *iucA* was higher and *iro* was lower than the results of the study by Xu et al. [26]. The *ybt* was the second most prevalent virulence factor gene among hvKp isolates in this study. The yersiniabactin gene and its receptor, which is an important virulence factor for the survival of *Klebsiella* strains under severe conditions, can transmit both an integrative conjugative element (ICEKp) and a plasmid (recently reported) [27]. Some studies have described the correlation between yersiniabactin-producing hvKps and pulmonary infectious diseases [28, 29]. In Iran, a study conducted by Tabrizi et al. reported that 5 of 53 K. *pneumoniae* strains isolated from ventilator-associated pneumonia were hvKp [30]. In the current study, of 33 hvKps isolated from lung-related samples, 27 isolates were *ybt*-positive, confirming the results of previous studies. The *rmpA* was identified as the fourth most virulence factor. Because *rmpA* increases the expression of capsular polysaccharide (CPS), we expected that the *rmpA*-producing hvKp that were isolated would be string test-positive, but this hypothesis was refuted by our results, such that only 36.7% of the *rmpA*-positive isolates were reported as hypercystic phenotype. Studies have shown that other genes besides *rmpA* are involved in capsular gene expression, such as regulation of capsular synthesis B (*rcsB*). Both *rmpA* and *rcsB* genes have been shown to co-occur [31]. In addition, the data show that the co-presence of four genes (*iucA*, *ybt*, *iutA* and *rmpA*) was more frequent in hvKp. In addition, other plasmid-born genes such as *iro*, *peg-344* were less frequent and were reported only sporadically.

Sequencing and analysis of large virulence plasmids from hvKp strains revealed that virulence-associated genes were mainly found in two regions. The *rmpA2*, *iucABCD* and *iutA* genes are located close to each other, followed by the *rmpA*, *peg-344* and *iroBCDN* genes in the second region. Some virulence plasmids carry all virulence genes (e.g. pLVPK, GenBank accession number: AY378100), but others have lost one or more virulence-associated loci, confirming our result (e.g. pVir, GenBank accession number: CP029383.2) [6, 32]. Despite most Asian countries having introduced K1 and K2 as the most common capsular serotypes [21, 33–35], we identified K20 as the most common capsular type in Iran. This phenomenon suggests that the prevalence of the different serotypes may vary depending on the geographical area. Although there has been no comprehensive study on the hvKp isolates in Iran and little information is available on them, no K1 and K2 were found among the *K. pneumoniae* isolates in the study conducted by Aghamohammad et al. [36]. Also, in another study, one K1 and 15 K2 were identified among 122 *K. pneumoniae* isolates from Semnan, Iran, which are in agreement with our results (we detected only one K1 and three K2) [37]. Another study from Iran conducted by Solgi et al. reported that the prevalence of K1 and K2 was 45.9% and 13.5% respectively, which was more than the present study [38].

Also, in the present study, according to the Table 3, in terms of frequency in the type of sample, and the hospital wards did not show a significant difference between these two variants. In both groups, almost half of the samples were isolated from the ICU. However, patients admitted to the ICU due to prolonged hospitalization were more susceptible to hvKp infection. In addition, it may increase the probability of horizontal gene transfer in clinical settings [39, 40]. In this study, it was also found that in-hospital B, most hvKps had the same genotypic characteristics such as capsular serotype K20, *ompK36* type C, and similar antibiotic resistance profiles. Therefore, the hvKp regional expansion hypothesis seems reasonable.

Most hvKps are sensitive to most antibiotics except for intrinsic resistance to ampicillin, similarly in this study all hvKp isolates were ampicillin resistant [40]. Studies have shown that hvKps are unlikely to take up DNA from other resistant bacteria due to the large size of the capsule and increased expression of capsule-related genes, therefore antibiotic resistance is less common in hvKps than in cKp isolates [41]. But contrary to expectations, the rate of resistance was not much different between the two variants hvKps and cKps. Our results are consistent with other studies that have shown that the rate of hvKp resistance is increasing worldwide [42, 43]. The current study revealed the high prevalence of MDR-hvKp and high resistance to imipenem (66%). Moreover, the presence of *bla*_TEM_, *bla*_SHV_, *bla*_CTX-M-15_, *bla*_OXA-48_ and *bla*_NDM-1_ was detected simultaneously in 56.8% of hvKp isolates. There was a significant difference between hvKps and cKps about carbapenem-resistance genes so that *bla*_OXA-48_ was more frequent in carbapenem-resistant hvKps, and in contrast, *bla*_NDM-1_ was more detected in carbapenem-resistant cKp isolates. The reason for this difference is not clear.

However, two pathways have been introduced for the emergence of MDR-hvKp strains, the horizontal acquisition of resistance genes by plasmids and mobile genetic elements (MGEs) by hvKp isolates (type I), and another pathway is the acquisition of the virulence-associated plasmid (e.g., pLVPK and pVir) by MDR-cKp (type II) [42, 44]. Ultimately, both mechanisms lead to the development of MDR-hvKp strains that are resistant to antibiotic treatment in addition to having a very high pathogenicity that poses a serious threat to public health.

The correlation of *ompK36* porin variants with specific sequence types (STs) of *K. pneumoniae* was first described by Papagiannitsis et al. *K. pneumoniae* isolates can be classified into four groups (designated groups A to D) by *ompK36* genotyping [13, 45]. There is a relationship between *ompK36* type and clonal group (CG). Also, some STs were reported to be associated with hvKp isolates, e.g. ST11 and ST23, that ST11 (CG258) belonged to *ompK36* group A and ST23 (CG23) belonged to *ompK36* group C [13, 46]. In this study, clonal relatedness by *ompK36* typing revealed that group C (70.6%) was the most common *ompK36* porin type among hvKp isolates. A study in Taiwan showed that *ompK36* group C was significantly more abundant among *K. pneumoniae* isolates [46]. This study was in agreement with our study in Iran.

## Conclusion

This study presented a new rapid screening method based on the resistance of hvKp to tellurite, which was superior than string test in phenotypic identification of hvKp isolates. The consideration of phenotypic detection along with genotyping of hvKp render a reliable identification of hypervirulent strains. In this study, a high prevalence of MDR-hvKp and a high level of resistance to imipenem (66%) were detected. In addition, co-existence of *bla*_TEM_, *bla*_SHV_, *bla*_CTX-M-15_, *bla*_OXA-48_ and *bla*_NDM-1_ was identified in 56.8% of hvKp isolates. Using the PCR-based *ompK36* typing method, which was simpler and less expensive than MLST, we were also able to investigate the clonal relatedness of the strains. It was found that the majority of hvKp isolates belonged to capsular serotype K20 and *ompK36* group C, which is related to CG23 (e.g. ST23). It seems that the expansion of MDR-hvKp in clinical settings is an inevitable event and this needs an urgent infection control program in the healthcare setting. The plasmids harboring virulence and antimicrobial resistance factors will change the clinical face of *K. pneumoniae* soon.

## Abbreviations

*K. pneumoniae*: *Klebsiella pneumoniae*
hvKp: Hypervirulent *Klebsiella pneumoniae*
*rmpA*: Regulator of mucoid phenotype
*magA*: Mucoviscosity-associated gene
*kfu*: *Klebsiella* Ferric uptake
*iroB*: Salmochelin iron uptake systems
*ybt*: Yersiniabactin
*allS*: Allantoin metabolism gene
AK: Amikacin
GN: Gentamicin
CTX: Cefotaxime
CAZ: Ceftazidime
CRO: Ceftriaxone
IMI: Imipenem
MRP: Meropenem
FEP: Cefepime
CIP: Ciprofloxacin
AMP: Ampicillin
AZM: Aztreonam
MIC: Minimum inhibitory concentration
ICU: Intensive care unit
ESBL: Extended spectrum beta-lactamases
pLVPK: Large Virulence Plasmid of *K. pneumoniae*
MDR: Multidrug resistant
MDR-cKp: Classical MDR-*K. pneumoniae*
ST: Sequence types
BAL: Bronchoalveolar lavage
CSF: Cerebrospinal fluid
